# A Proline/Arginine-Rich End Leucine-Rich Repeat Protein (PRELP) Variant Is Uniquely Expressed in Chronic Lymphocytic Leukemia Cells

**DOI:** 10.1371/journal.pone.0067601

**Published:** 2013-06-24

**Authors:** Eva Mikaelsson, Anders Österborg, Mahmood Jeddi-Tehrani, Parviz Kokhaei, Mahyar Ostadkarampour, Reza Hadavi, Mehran Gholamin, Mehdi Akhondi, Fazel Shokri, Hodjattallah Rabbani, Håkan Mellstedt

**Affiliations:** 1 Immune and Gene Therapy Laboratory, Cancer Centre Karolinska, Department of Oncology-Pathology, Karolinska Institutet, Stockholm, Sweden; 2 Departments of Hematology and Oncology, Karolinska University Hospital Solna, Stockholm, Sweden; 3 Monoclonal Antibody Research Center, Avicenna Research Institute, ACECR, Tehran, Iran; 4 Department of Immunology, Semnan University of Medical Sciences, Semnan, Iran; 5 Human Genetic Lab, Immunology Research Center, School of Medicine, Mashhad University of Medical Sciences, Mashhad, Iran; 6 Reproductive Biotechnology Research Center, Avicenna Research Institute, ACECR, Tehran, Iran; 7 Department of Immunology, School of Public Health, Tehran University of Medical Sciences, Tehran, Iran; University of Thessaly, Faculty of Medicine, Greece

## Abstract

Proline/arginine-rich end leucine-rich repeat protein (PRELP) belongs to the small leucine-rich proteoglycan (SLRP) family, normally expressed in extracellular matrix of collagen-rich tissues. We have previously reported on another SLRP, fibromodulin (FMOD) in patients with chronic lymphocytic leukemia (CLL). PRELP is structurally similar to FMOD with adjacent localization on chromosome 1 (1q32.1). As cluster-upregulation of genes may occur in malignancies, the aim of our study was to analyze PRELP expression in CLL. PRELP was expressed (RT-PCR) in all CLL patients (30/30), as well as in some patients with mantle cell lymphoma (3/5), but not in healthy donor leukocytes (0/20) or tumor samples from other hematological malignancies (0/35). PRELP was also detected in CLL cell-lines (4/4) but not in cell-lines from other hematological tumors (0/9). PRELP protein was detected in all CLL samples but not in normal leukocytes. Deglycosylation experiments revealed a CLL-unique 38 kDa core protein, with an intact signal peptide. This 38 kDa protein was, in contrast to the normal 55 kDa size, not detected in serum which, in combination with the uncleaved signal peptide, suggests cellular retention. The unique expression of a 38 kDa PRELP in CLL cells may suggest involvement in the pathobiology of CLL and merits further studies.

## Introduction

The pathobiology of chronic lymphocytic leukemia (CLL) has become an increasingly explored area of research. In addition to understanding the role of the microenvironment, one of the major goals has been to identify genes involved in the pathogenesis of the disease. In 2001, gene expression profiling revealed, among others, fibromodulin (FMOD) as one of the most overexpressed genes in CLL compared to memory B cells of healthy donors.[1] FMOD is a member of the small leucine-rich proteoglycan family (SLRP) and is normally expressed in collagen-rich tissues. We demonstrated that FMOD was expressed at the gene and protein level in CLL and mantle cell lymphoma (MCL).[2] This unexpected finding of an aberrantly expressed extracellular matrix protein raised the question whether also other SLRP family members might be expressed in CLL.

Overexpression of genes in tumor cells might be due to epigenetic regulations, which may span a cluster of closely located genes. The proline/arginine-rich end leucine-rich repeat protein (PRELP) is structurally similar to FMOD and is located about 80 kb 3′-proximal to FMOD on chromosome 1q32.1.[3] Human PRELP has been reported to have a molecular weight (MW) of 55 kDa and is normally expressed in the extracellular matrix of connective tissues, preferentially in cartilage, lung, kidney, skin, and tendon.[4], [5] The function of PRELP is unclear, but the interactions between PRELP and collagen type I and II as well as heparin and heparan sulphate[6], [7] suggest that PRELP may be a molecule anchoring basement membranes to connective tissue.[7]

Following our previous studies on FMOD[2] and ROR1[8] in CLL, both located on chromosome 1, the present study was undertaken to explore the gene and protein expression of PRELP in CLL and other hematological malignancies, in our endeavour to explore uniquely expressed molecules in CLL which may play a role in the pathobiology of the disease.

## Materials and Methods

### Patients and controls

Diagnosis of CLL and other hematological malignancies was established using the WHO classification of hematopoetic and lymphoid malignancies and the modified NCI criteria.[9] Clinical characteristics of the CLL patients are shown in Table 1. Progressive and non-progressive CLL was defined as recommended by the IWCLL criteria.[10]

**Table 1:** Clinical characteristics of the CLL patients (n = 30).

Characteristic		Frequency (%)
Sex	Male	72
	Female	28
Age, years	40–49	7
	50–59	7
	60–69	36
	70–79	40
	80–89	10
Clinical phase	Progressive*	47
	Non-progressive*	53
Rai Stage	O	37
	I	27
	II	7
	III	27
	IV	3
Treatment	Previously treated**	33
	Untreated***	67

*for definition, see ref [10].

**test performed at relapse/progression prior to initiation of salvage therapy.

***Among 20 untreated patients, 11 were indolent and 9 were progressive/symptomatic but tested prior to first-line therapy.

Heparinized blood containing tumor cells was collected from patients with CLL (n = 30), MCL (n = 5), hairy cell leukemia (HCL) (n = 2), B-cell prolymphocytic leukemia (B-PLL) (n = 2), T-cell prolymphocytic leukemia (T-PLL) (n = 4), chronic myelogenous leukemia (CML) (n = 5), acute myelogenous leukemia (AML) (n = 5) and acute lymphoblastic leukemia (ALL) (n = 10). Bone marrow tumor cells were obtained from patients with multiple myeloma (MM) (n = 6), and follicular lymphoma (FL) (n = 2). Blood was also drawn from healthy control donors (n = 20). Serum was collected from CLL patients (n = 8) and healthy controls (n = 8).

All samples were collected with written informed consent of the patients and approval from the regional ethics committee (The regional ethical review board in Stockholm, www.epn.se).

### Hematological cell lines

Four CLL cell lines and nine cell lines derived from a variety of other hematological malignancies were also studied; CLL (EHEB, I83-E95, 232-B4, WAC3-CD5), MM (LP-1), T-cell leukemia (SKW3), ALL (HUT-78, HPB-ALL, MOLT-4, JURKAT), AML (HL60), CML (K562), and NK cell lymphoma (YT). EHEB and YT were obtained from DSMZ (Braunschweig, Germany). The other CLL cell lines (I83-E95, 232-B4, WAC3-CD5)[11], [12] were a kind gift from Prof. Anders Rosén (Linköping University, Sweden) and Prof. Kenneth Nilsson (Uppsala University, Sweden). The remaining cell lines were provided by the National Cell Bank of Iran (NCBI, Pasteur Institute of Iran, Tehran, Iran). All cell lines were adapted to grow in RPMI-1640 medium supplemented with 10% fetal bovine serum (FBS), L-glutamine (2 mM), penicillin (100 U/ml) and streptomycin (100 µg/ml) (Gibco, Paisley, Scotland).

### Isolation of cells

Peripheral blood mononuclear cells (PBMC) from normal donors and leukemic cells from blood and bone marrow were isolated using Ficoll-Paque Plus (GE Healthcare, Buckinghamshire, UK) density-gradient centrifugation. Normal T and B lymphocytes were purified be negative selection using MACS beads (Miltenyi Biotec, Bergisch Gladbach, Germany) according to the manufacturer's instructions. For isolation of CLL T-cells, the CLL PBMCs were depleted by filtration through a nylon wool column (Biotest, Breiech, Germany). The effluent cells were further enriched by negative selection using MACS beads. The purity of the isolated populations was tested by direct immunofluorescence using monoclonal antibodies against CD3, CD19, and CD14 (BD Biosciences, San Jose, CA, USA). The purity was >90% for normal B cells and >70% for normal T cells, and >80% for CLL T-cells. Granulocytes were recovered from the top of the erythrocyte layer. Erythrocytes were lysed by hypo-osmosis in cold water. More than 98% of the nucleated cells were granulocytes as evaluated by immunocytology.

### RT-PCR and quantitative PCR (q-PCR) amplification of PRELP mRNA

Total RNA was extracted from tumor cells and normal PBMC using PureLink™ RNA Mini Kit (Ambion, Carlsbad, CA, USA) according to the manufacturer's instruction. First strand cDNA was synthesized using SuperScript™ III Reverse Transcriptase (Invitrogen, Carlsbad, CA, USA) according to the manufacturer's protocol.

PCR amplification was performed using PRELP specific primers (Table 2). Briefly, 25 µl of PCR reaction mixture was prepared using 2.5 µl of 10× buffer, 2 µl of 25 mM MgCl_2_, 1.5 µl dNTPs (10 mM), 5 pmol of each primer and 1 unit of Ampli-Taq Gold DNA polymerase (Perkin-Elmer/Applied Biosystems, Boston, MA, USA). PCR was initiated by 1 cycle at 95°C for 10 min, followed by 35 cycles of 92°C; 30 sec, 60°C; 30 sec, and 72°C; 30 sec leading to a 334 bp amplicon. To assure the specificity of primers, some PCR products were cloned into pGEM-T easy vector (Promega, Madison, WI, USA) and subjected to sequencing.

**Table 2:** Primers used in RT-PCR amplification of PRELP.

Target	Primer (5′→3′)	Position	Amplicon (bp)	Reference
PRELP	S: TCAAGAACCTCATGCAGCTCAA	777–798	334	g.b. NM_002725
	AS: ATCTGGGTTCCGTTGATTTTCTC	1088–1110		
Beta-actin	S: ATTAAGGAGAAGCTGTGCTACGTC	707–730	215	g.b. NM_001101
	AS: ATGATGGAGTTGAAGGTAGTTTCG	898–921		

S  =  Sense, AS  =  Antisense, g.b  =  genebank.

Quantitative q-PCR was performed using Taqman gene expression assay for PRELP (Applied Biosystems, assay ID# Hs00160431_m1). PCR reactions consisting of 1 µL cDNA, 1X TaqMan® Universal PCR Master Mix, 0.2 µM TaqMan® primer-probe mix were carried out in final volumes of 20 µL using an ABI Prism 7900 Sequence Detection System (Applied Biosystems). Reactions were initiated with a 10 minute incubation at 95°C followed by 40 cycles of 95°C for 15 seconds and 60°C for 60 seconds. The analysis was based on individual samples run in triplicates, of which a mean Ct value was calculated. The expression of PRELP was then normalized against the housekeeping gene GAPDH (Applied Biosystem assay #Hs02758991_g1) and relative quantities were determined by the comparative Ct (ΔCt) method.

### Production of PRELP protein

Due to lack of commercially available PRELP protein, we established a yeast expression system based on cDNA from PBMC of CLL patients (n = 10) that were pooled, and a full-length PRELP transcript was PCR-amplified. The PCR product was cloned into pGEM-T easy vector and subcloned into pGAPZα-A vector for yeast *P. pastoris* (Invitrogen). The recombinant plasmids were selected for sequencing. After selecting an in-frame clone, the construct was linearized using *Avr*II restriction enzyme and transfected into *P. pastoris* strain SMD1168 (Invitrogen). The colonies were screened by gene specific PCR amplification and positive clones were selected for protein production. The supernatant of a 72 h cultured yeast clone was collected and concentrated up to 30 times using Amicon Ultra-15 Centrifugal Filter Units (Millipore Corporation, Bedford, MA, USA).

For expression in mammalian cells, a full-length PRELP cDNA clone (transcript variant 1, SC111673, TrueClones, OriGene Technologies, Inc. Rockville, MD, USA) was subcloned into NotI site of a mammalian expression vector pCMV6-Neo (OriGene Technologies). After selection and sequencing of an in-frame clone, the plasmid was transfected into mouse SP2/0 cell line to obtain stable transfectants using jetPEI™ transfection reagent (Polyplus-transfection™, Illkirch, France). Cells were harvested, washed extensively and lysate prepared as described for Western blot.

### Chemical deglycosylation of PRELP protein

Recombinant PRELP protein produced in yeast was subjected to chemical deglycosylation using trifluoromethanesulfonic acid (TFMS) (Sigma, St Louis, MO, USA) and anisole (Fluka, Sigma). TFMS removes all carbohydrates chains from glycoproteins regardless of linkage and composition.[13]

250 µl of yeast culture supernatant was precipitated in 100% ethanol at −20°C over night in two separate tubes. Protein pellets were collected by centrifugation at 15000 g for 20 min, washed in 95% ethanol, collected by centrifugation and air-dried for 1 h. 200 µl TFMS and anisole (9∶1) was added to the dry pellets and the samples were incubated on ice for 2 and 4 h, respectively. The reaction was stopped by the addition of 2 M Tris base (pH 8) until pH reached 6. The samples were dialysed against 10 mM phosphate buffer for 24 h, concentrated 20 times in Amicon Ultra-15 Centrifugal Filter Units (Millipore Corp.) and then subjected to Western blot.

### Anti-PRELP poly- and monoclonal antibodies

In the absence of commercially available anti-PRELP antibodies recognizing PRELP in CLL, one rabbit anti-PRELP polyclonal antibody (pAb) was produced against a 19-mer peptide (CGGKARAKGGFRLLQSVVI) purchased from Thermo Electron Corporation GmbH (Ulm, Germany) of which the 9 last amino acids correspond to the carboxy-terminal (C-terminal) part of human PRELP.[4] The antibody was purified by affinity chromatography.

In addition, two mouse anti-PRELP monoclonal antibodies (mAbs) were produced using Keyhole limpet hemocyanin (KLH)-conjugated PRELP-peptides following a standard protocol with minor modifications.[14] One mouse mAb was generated against the carboxy-terminal peptide (CGGKARAKGGFRLLQSVVI). The other mAb was raised against the N-terminal region for which a 20-mer peptide (MRSPLCWLLPLLILASVAQG) (Thermo Electron) covering the whole signal sequence was used.

### Western blot

Cell lysates were prepared as described with minor modifications.[15] Briefly, 50×10^6^ cells were lysed in 1 mL of buffer containing 0.2% triton-X, 130 mM HEPES, 4 mM MgCl_2_, 10 mM EGTA with 2% proteinase inhibitor cocktail (Sigma). After 1 h incubation on ice, lysates were centrifuged at 2500 rpm for 5 min and the soluble fraction was collected. The protein concentration was measured by Bio-Rad Protein Assay according to the manufacturer's instructions (Bio-Rad Laboratories, Hercules, CA, USA). Cell lysate (20 µg), serum (dil 1∶50), and yeast supernatants were subjected to Western blot using a 10% NuPAGE Bis-Tris gel (Invitrogen) at 120 V for 3 h under reducing conditions. Resolved proteins were transferred onto Immobilon-P PVDF membrane (Millipore Corp.) in a Mini-Transblot Cell (Invitrogen). Non-specific antibody binding was blocked by incubating the membranes at room temperature for 1,5 h with 5% non-fat milk (Semper, Stockholm, Sweden) in PBS plus 0.05% Tween 20 (PBS-T). The membranes were incubated with 10 µg/ml of anti-PRELP rabbit polyclonal or mouse monoclonal antibody over night at +4°C and a secondary horseradish peroxidise (HRP)-conjugated goat anti-rabbit or rabbit anti-mouse antibody (DakoCytomation, Glostrup, Denmark) for 1.5 h at room temperature. 4×15 min washings in PBS-T followed both incubations. Antibody-reactive bands were visualized using Amersham Enhanced Chemiluminescence ECL™ system (GE Healthcare). To verify equal loading of samples, filters were stripped in a buffer containing 62.5 mM Tris-HCL, 2% SDS, 100 mM Mercaptoethanol (Sigma) at 50°C for 30 min. Following 3×15 min washing in PBS-T, the membranes were re-probed with 2.5 µg/ml of a mouse anti-β-actin monoclonal antibody (Sigma).

### Statistical analysis

The results are shown as mean ± SEM and statistical analysis was performed with a T-test or Anova multivariant analysis together with post hoc Tukey test.

## Results

### PRELP gene expression

**Figure 1:**
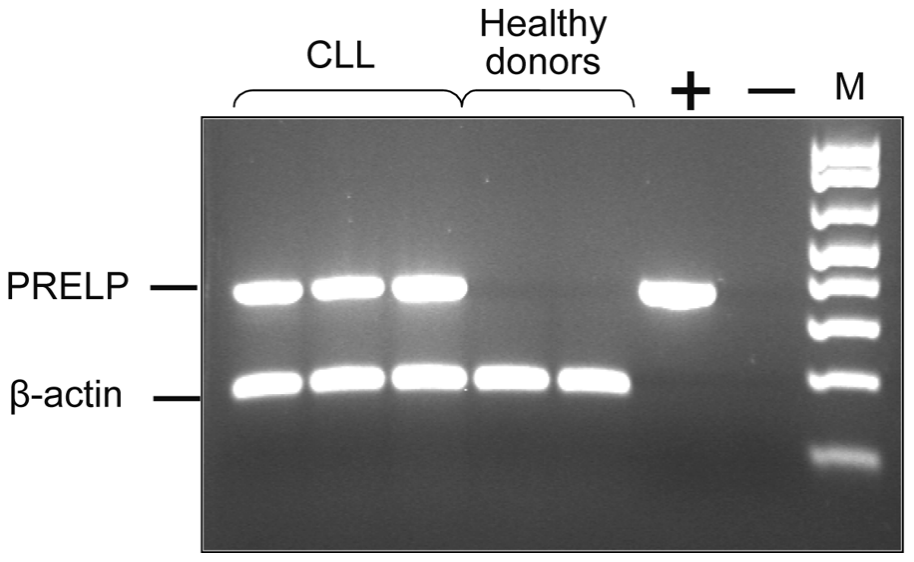
PRELP gene expression (RT-PCR) in PBMC of CLL patients and healthy control donors. Three CLL patients (lane 1–3) and 2 healthy controls (lane 4–5) were tested. Positive (+) control represents PCR product cloned into pGEM-T easy vector. Negative (−) control is the reaction mix without template. Marker (M) is a 100-bp DNA ladder.

The expression of PRELP mRNA was tested by RT-PCR in tumor cells from CLL patients and other hematological malignancies as well as in PBMC and leukocyte subsets from healthy control donors. First, we tested PRELP mRNA expression in PBMC from CLL patients and healthy donors. PBMC from all CLL patients (n = 30) expressed PRELP at the mRNA level irrespective of clinical phase (non-progressive/progressive) (Figure 1). PRELP mRNA was not expressed in isolated CLL T-cells (0/10). Neither was PRELP expressed in fresh PBMC (lymphocytes and monocytes) of healthy donors (0/20), enriched normal blood B cells (0/6), T cells (0/4), or granulocytes (0/5). The results are summarized in Supplementary Table S1. Sequencing of cDNA from CLL patients (n = 10) revealed no major mutations in the PRELP gene (data not shown).

Next, we tested PRELP mRNA expression in other hematological malignancies. PRELP was expressed in tumor cells of some MCL patients (3/5) but not in FL (0/2), T- or B-PLL (0/6), HCL (0/2), MM (0/6), CML (0/5), AML (0/5), and ALL (0/10) (Supplementary Table S1).

Thereafter, we tested cell lines and found that PRELP mRNA was expressed in all four CLL cell lines (EHEB, I83-E95, 232-B4, WAC3-CD5) but not in cell lines derived from myeloma (0/1), T cell leukemia (0/1), ALL (0/4), AML (0/1), CML (0/1), and NK cell lymphoma (0/1) (Supplementary Table S2).

**Figure 2:**
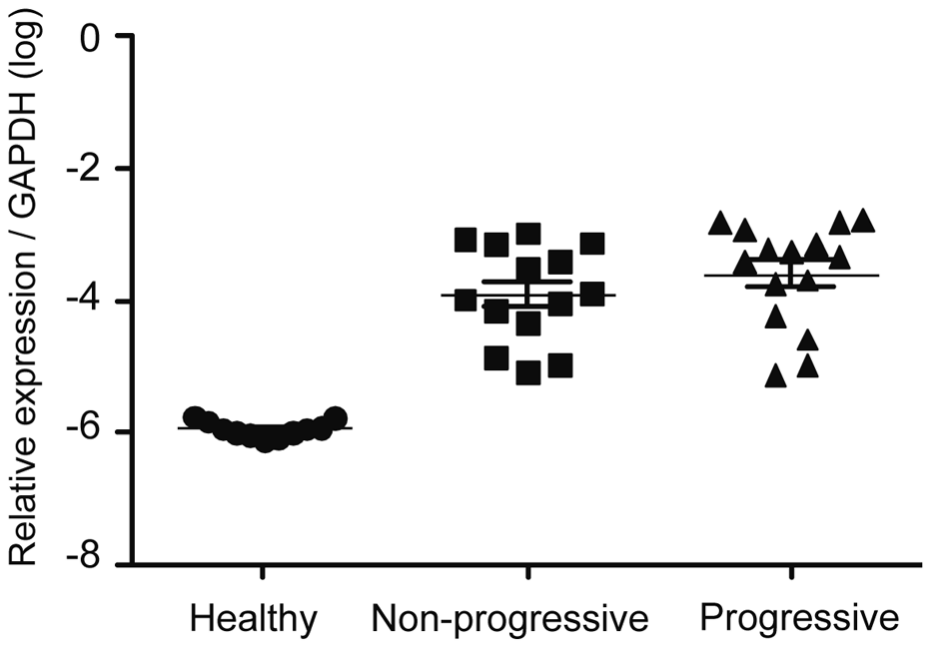
Quantitative PRELP gene expression (q-PCR) in PBMC of CLL patients and healthy control donors. The relative expression of PRELP was significantly higher in CLL patients (n = 30) compared to healthy control donors (n = 12) (p<0,0001). Dividing the CLL samples into progressive and non-progressive resulted in significantly higher relative expression of PRELP in both progresssive and non-progressive compared to healthy donors (p<0,001). There was no difference in PRELP expression between non-progressive and progressive CLL. Relative expression of PRELP was calculated using the ΔCt method and GAPDH as reference gene.

By q-PCR, the expression of PRELP mRNA was quantified in relation to the housekeeping gene GAPDH. The relative expression of PRELP was significantly higher in CLL patients (n = 30) compared to healthy control donors (n = 12) (p<0,0001). Also, dividing the CLL samples into progressive and non-progressive resulted in higher relative expression of PRELP in both groups compared to healthy donors (p<0,001 respectively). There was no difference in PRELP expression between progressive and non-progressive CLL (Figure 2).

### Specificity of anti-PRELP antibodies

**Figure 3:**
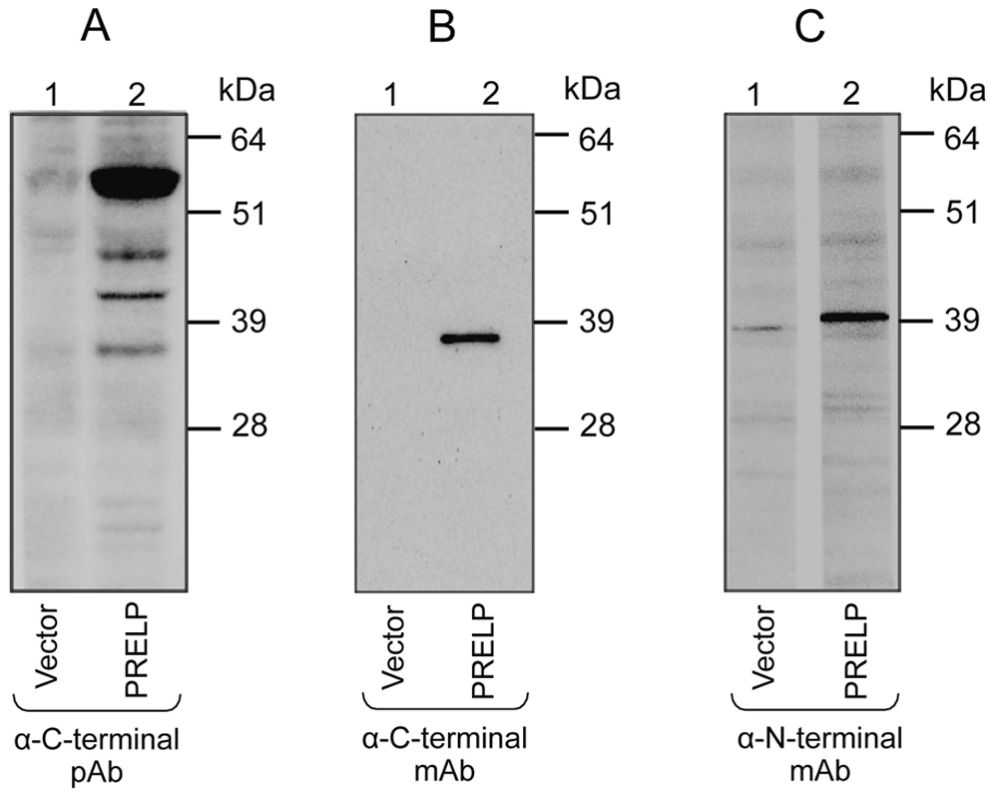
Western blot analyses to validate the specificity of own-produced anti-PRELP antibodies. The precursor PRELP gene was cloned into pCMV6-Neo vector and transfected into mouse SP2/0 cell line. **Lane 1:** Cell lysate of SP2/0 cells transfected with the vector alone (negative control). **Lane 2:** Cell lysate of SP2/0 cells transfected with the PRELP construct. Recombinant PRELP was detected as follows: **A)** A rabbit anti- C-terminal PRELP length glycosylated PRELP.[4] Smaller bands were also obtained (38, 44 and 48 kDa) **B)** mouse monoclonal antibody (mAb) against C-terminal PRELP recognized a 38 kDa band only **C)** same finding for a mouse mAb against N-terminal PRELP.

The specificity of our own-produced anti-PRELP poly- and monoclonal antibodies was tested against recombinant PRELP protein expressed in SP2/0 mouse cell line (Figures 3A–C). Cells transfected with pCMV6-Neo vector alone were used as a negative control. The MW of normal, full-length glycosylated PRELP protein is 55 kDa.[4] In Western blot, the C-terminal rabbit polyclonal anti-PRELP antibody recognized a major band of 55–58 kDa but also three additional bands of 38, 44, and 48 kDa respectively. The mouse monoclonal anti-PRELP antibodies (C-terminal as well as N-terminal) recognized a 38 kDa PRELP band only, suggesting different epitope binding by our panel of own-produced pAb and mAb against PRELP.

### PRELP protein expression in CLL cells

**Figure 4:**
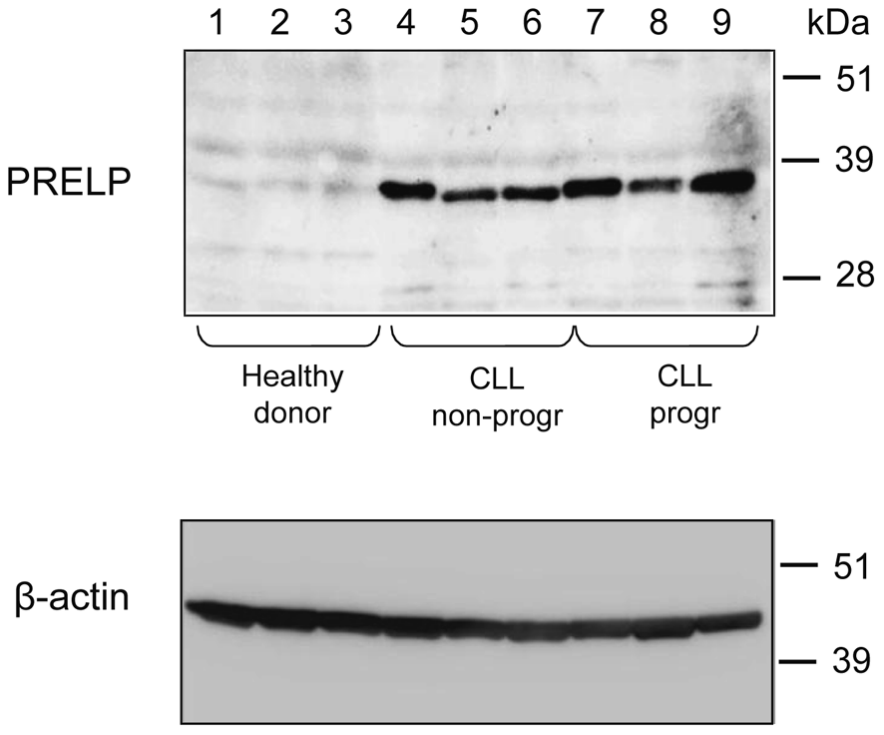
Western blot of cell lysates from CLL patients and healthy control donor PBMC. Three healthy controls (lane 1–3), 3 non-progressive CLL (lane 4–6) and 3 progressive CLL patients (lane 7–9) were tested. *Upper panel*: the rabbit anti- C-terminal PRELP polyclonal antibody detected a 38 kDa band in CLL cells but not in healthy donor PBMC. *Lower panel*: The same membrane stripped and re-probed with an anti-β-actin monoclonal antibody.

Having confirmed the PRELP specificity of our anti-PRELP pAb and mAb, we thereafter used PBMC from CLL patients (n = 30) and analysed PRELP protein expression by Western blot. In tumor cell lysates, a band of 38 kDa was detected in all CLL patients and all four CLL cell lines. This 38 kDa band was recognized both by the C-terminal and the N-terminal antibodies eliminating the possibility that the 38 kDa fragment is a degradation product. In contrast, the PRELP protein was not detected in PBMC of healthy control donors (n = 10) (Figure 4).

### Deglycosylation of the PRELP protein

**Figure 5:**
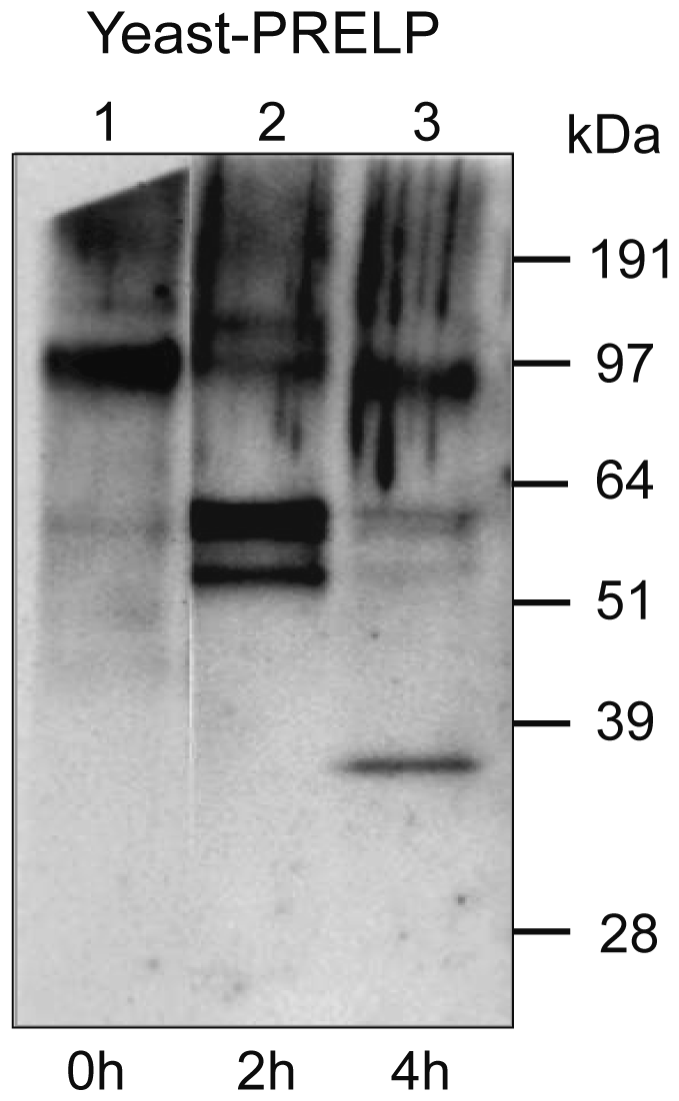
Chemical deglycosylation by TMFS of a yeast-derived recombinant mature PRELP protein. Western blot was performed using the anti- C-terminal polyclonal antibody. **Lane 1**: Untreated (0 h) yeast-derived recombinant PRELP showing a 100 kDa band. **Lanes 2 and 3**: Yeast-derived recombinant PRELP treated with TFMS for 2 and 4 h, respectively. After complete removal of the carbohydrate structures, a 38 kDa band was seen.

Unmodified yeast-derived PRELP had a MW of about 100 kDa (Figure 5). To explore the nature of the various PRELP bands found in our initial experiments (Figure 3A–C), we performed chemical deglycosylation using TFMS and anisole. The results are shown in Figure 5. After 2 h of treatment, bands in the region of 51–64 kDa appeared. After 4 h, a band of 38 kDa was seen, probably representing a completely deglycosylated PRELP protein (which is in line with the 38 kDa size band found in CLL cells and CLL cell lines shown in Figure 4).

### PRELP protein expression in serum

**Figure 6:**
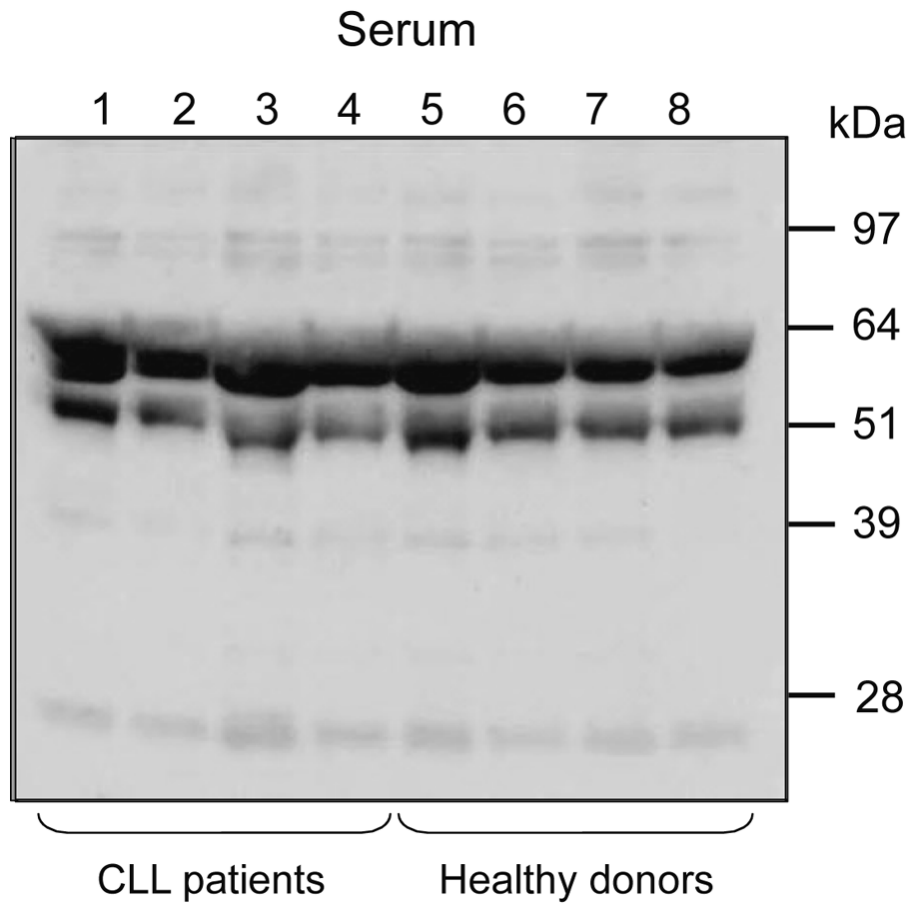
Western blot of serum samples (1∶50) from CLL patients and healthy donors. Four CLL patients (lane 1–4) and 4 healthy controls (lane 5–8) were tested. The rabbit anti- C-terminal PRELP polyclonal antibody detected 50 kDa and 58 kDa PRELP bands in all samples, but no band was seen at 38 kDa.

We also analyzed serum from 8 CLL patients and 8 healthy control donors by Western blot. All serum samples (both CLL and healthy controls) showed two bands, 50 and 58 kDa (Figure 6), corresponding to full-length PRELP.[4] The 38 kDa PRELP protein was not detected in serum from either patients or normal donors.

## Discussion

The present study demonstrates that the SLRP family member PRELP seems to be exclusively expressed in CLL cells and CLL cell lines as well as in some patients with MCL. Other haematological malignancies and cell lines as well as PBMC of normal donors did not express PRELP. Strong polyclonal activation (PMA/ionomycin) of normal B and T lymphocytes did not induce PRELP expression (data not shown), suggesting that the expression in CLL might reflect a constitutive tumor-related aberration *in vivo*.

We related the expression of PRELP to age, gender, leukemic cell count, genomic aberrations (13q del, 17p del, trisomy12) and previous treatment but no relation could be found. Also, RAI stage and CD38 expression analysed in a subset of patients showed no correlation to PRELP expression (data not shown). Zap-70 and IgHV mutational status were not tested.

PRELP is normally secreted into the extracellular matrix compartment but its exact function is not clearly known. The 50 and 58 kDa PRELP proteins detected in serum from both CLL patients and healthy controls may correspond to mature glycosylated PRELP[4], and might be produced by fibroblasts in the connective tissues. However, in the CLL cell lysates, a unique 38 kDa PRELP protein was identified. This 38 kDa protein was detected by both C-terminal and N-terminal anti-PRELP antibodies suggesting that the 38 kDa protein is not a cleavage- or degradation product. This is further supported by deglycosylation experiments that reduced all PRELP bands with the exception for the 38 kDa band. Mutation analysis of the PRELP gene in CLL did not reveal any substantial nucleotide aberrations. These findings, in combination with only two coding exons, make splice variants or truncation unlikely.

The own produced anti-PRELP N-terminal antibody was directed against the signal peptide and recognized a 38 kDa CLL-specific PRELP band, indicating that the signal peptide was not cleaved off. The presence of an intact signal peptide may suggest retention of the 38 kDa PRELP in the cytosol. This is further supported by the finding that the 38 kDa PRELP protein was not detected in serum and thus probably not secreted by CLL cells. Rapid degradation of the 38 kDa PRELP in serum by proteases could however not be excluded.

Recombinant PRELP expressed in SP2/0 cells, was detected by our polyclonal C-terminal antibody as weak bands of 38, 44, 48 kDa and a major band of 55–58 kDa. This is assumed to represent varying degrees of glycosylation. Our anti-PRELP mAbs, recognized however only the 38 kDa band. An explanation might be that our mAbs recognized epitopes that are hidden in the mature PRELP three-dimensional folding.

This is the first report connecting PRELP with CLL (and partly to MCL). There are reports linking other SLRPs to cancer.[16], [17] We have previously reported on the unique CLL expression of the closely related SLRP FMOD.[2] SLRPs are normally secreted proteins that mediate their functions by binding to membrane receptors or extracellular matrix proteins. However, other locations and functions have been reported. PRELP has been shown to bind and inhibit NF-kappa B activity in the nucleus of osteoclasts.[18]

Our findings suggest the presence of a predominant, non-secreted unglycosylated 38 kDa variant of PRELP in CLL cells. The specific expression of another structurally related proteoglycan, FMOD[2], in CLL as well as a third SLRP, opticin (OPTC) (own unpublished observation), located in close proximity to FMOD and PRELP on chromosome 1 (1q32) may suggest a broader role of proteoglycans in CLL. Functional characterization of proteoglycans in CLL is warranted to understand their biological importance in the pathogenesis of CLL.

## Supporting Information

Table S1 **PRELP gene expression (RT-PCR) in PBMCs from patients with various types of hematological malignancies and normal leukocyte subsets of healthy control donors.**
(DOCX)

Table S2 **PRELP gene expression (RT-PCR) in hematological cell lines.**
(DOCX)
