# Supplementary material for: A Proline/Arginine-Rich End Leucine-Rich Repeat Protein (PRELP) Variant Is Uniquely Expressed in Chronic Lymphocytic Leukemia Cells
Source: PLoS One. 2013 Jun 24;8(6):e67601. doi: 10.1371/journal.pone.0067601 (PMC3691130; doi:10.1371/journal.pone.0067601)
Supplement: Table S1 — PRELP gene expression (RT-PCR) in PBMCs from patients with various types of hematological malignancies and normal leukocyte subsets of healthy control donors. (DOCX) [file pone.0067601.s001.docx]

**Table S1.** PRELP gene expression (RT-PCR) in PBMCs from patients with various types of hematological malignancies and normal leukocyte subsets of healthy control donors

| **Cell source** | **No. of positive cases/total no.** |
| --- | --- |
| Chronic lymphocytic leukemia (PBMC) | 30/30 |
| Chronic lymphocytic leukemia (purified T cells) * | 0/10 |
| Mantle cell lymphoma (PBMC) | 3/5 |
| Chronic myelogenous leukemia (PBMC) | 0/5 |
| Acute lymphoblastic leukemia (PBMC) | 0/10 |
| Acute myelogenous leukemia (PBMC) | 0/5 |
| Prolymphocytic leukemia (B and T cell types) (PBMC) | 0/6 |
| Hairy cell leukemia (PBMC) | 0/2 |
| Follicular lymphoma (BMMC) | 0/2 |
| Multiple myeloma (BMMC) | 0/6 |
| Normal healthy PBMC (lymphocytes and monocytes) | 0/20 |
| Normal blood T cells** | 0/4 |
| Normal blood B cells*** | 0/6 |
| Normal blood granulocytes**** | 0/5 |

PBMC; peripheral blood mononuclear cells, BMMC; bone marrow mononuclear cells

*Purity > 80%, **Purity >70%, *** Purity >90%, ****purity >98%
