# Supplementary material for: A Proline/Arginine-Rich End Leucine-Rich Repeat Protein (PRELP) Variant Is Uniquely Expressed in Chronic Lymphocytic Leukemia Cells
Source: PLoS One. 2013 Jun 24;8(6):e67601. doi: 10.1371/journal.pone.0067601 (PMC3691130; doi:10.1371/journal.pone.0067601)
Supplement: Table S2 — PRELP gene expression (RT-PCR) in hematological cell lines. (DOCX) [file pone.0067601.s002.docx]

**Table S2.** PRELP gene expression (RT-PCR) in hematological cell lines

| **Malignancy** | **Cell line** | **PRELP expression** |
| --- | --- | --- |
| CLL | EHEB | Positive |
| CLL | I83-E95 | Positive |
| CLL | 232-B4 | Positive |
| CLL | WAC3-CD5 | Positive |
| Multiple myeloma | LP-1 | Negative |
| T cell leukemia | SKW3 | Negative |
| Acute lymphoblastic leukemia | HUT-78 | Negative |
| Acute lymphoblastic leukemia | HPB-ALL | Negative |
| Acute lymphoblastic leukemia | MOLT-4 | Negative |
| Acute lymphoblastic leukemia | JURKAT | Negative |
| Acute myelogenous leukemia | HL60 | Negative |
| Chronic myelogenous leukemia | K562 | Negative |
| NK lymphoma | YT | Negative |
